# Serological testing for syphilis in the differential diagnosis of cognitive decline and polyneuropathy in geriatric patients

**DOI:** 10.1186/s12877-023-03981-4

**Published:** 2023-05-05

**Authors:** Marija Djukic, Helmut Eiffert, Peter Lange, Ioanna Giotaki, Jana Seele, Roland Nau

**Affiliations:** 1 Institute of Neuropathology, Universitätsmedizin Göttingen, Göttingen, Germany; 2 Department of Geriatrics, Protestant Hospital Göttingen-Weende, An der Lutter 24, Göttingen, 37075 Germany; 3 Amedes MVZ for Laboratory Medicine, Medical Microbiology and Infectiology, Göttingen, Germany; 4 Department of Neurology, Universitätsmedizin Göttingen, Göttingen, Germany

**Keywords:** Neurosyphilis, Electrochemiluminescence immunoassay, Immunoblot, Cerebrospinal fluid, Treponema-specific antibody index

## Abstract

**Background:**

In the 19th century, neurosyphilis was the most frequent cause of dementia in Western Europe. Now dementia caused by syphilis has become rare in Germany. We studied whether routine testing of patients with cognitive abnormalities or neuropathy for antibodies against Treponema pallidum has therapeutic consequences in geriatric patients.

**Methods:**

A Treponema pallidum electrochemiluminescence immunoassay (TP-ECLIA) is routinely performed in all in-patients treated at our institution with cognitve decline or neuropathy and no or insufficient previous diagnostic workup. Patients with a positive TP-ECLIA treated from October 2015 to January 2022 (76 months) were retrospectively evaluated. In cases of positive TP-ECLIA, further specific laboratory investigations were performed to assess whether antibiotic therapy was indicated.

**Results:**

In 42 of 4116 patients (1.0%), TP-ECLIA detected antibodies directed against Treponema in serum. Specifity of these antibodies was ensured by immunoblot in 22 patients (11 × positiv, 11 × borderline values). Treponema-specific IgM was detectable in the serum of one patient, in 3 patients the Rapid Plasma Reagin (RPR) test, a modified Venereal Disease Research Laboratory test (VDRL), in serum was positiv. CSF analysis was performed in 10 patients. One patient had CSF pleocytosis. In 2 other patients, the Treponema-specific IgG antibody index was elevated. 5 patients received antibiotic therapy (4 × ceftriaxone 2 g/d i.v., 1 × doxycycline 300 mg/d p.o.).

**Conclusion:**

In approx. 1‰ of patients with previously undiagnosed or not sufficiently diagnosed cognitive decline or neuropathy, the diagnostic workup for active syphilis resulted in a course of antibiotic treatment.

## Introduction

Cognitive decline and neuropathy are two key symptoms of tertiary syphilis. In the 19th century and the first half of the 20th century, neurosyphilis world-wide was the most frequent cause of dementia. Syphilis was very frequent, there was no adequate treatment for early syphilis, and life expectancy was low, i.e., degenerative dementias were much rarer than today [1, 2]. As a consequence of penicillin treatment, syphilis infections and syphilis-caused dementia have become rare in developed countries [3]. Traditionally, laboratory tests on the differential diagnosis of dementia included syphilis serology in addition to a complete blood count, electrolyte and metabolic screen, thyroid panel, vitamin B12 and folate levels, urinalysis, chest radiograph and electrocardiogram, and head CT scan [4]. In newer guidelines, syphilis serology is not considered mandatory for the differential diagnosis of dementia, but is recommended “in individual cases” not further specified (e.g., [2, 5, 6]).

Dementia is a symptom of late (“tertiary”) forms of syphilis. It may occur in meningovascular neurosyphilis (often occurring from 5 to 12 years after infection), but the most frequent cause of syphilitic dementia is general paresis (usually occurring from 10 to 25 years after infection). Neuropathy is a typical symptom of tabes dorsalis occurring 15 to 25 years after infection [2, 7]. In the last decades, after the introduction of antibiotic treatment, many cases of neurosyphilis with symptoms not fitting into the classical forms of secondary and tertiary syphilis have been published (e.g. [7–12]). Probably as a consequence of antibiotic treatment courses for other indications with partial effectivity also against Treponema pallidum (TP), atypical forms, which cannot be categorized into one of the classical forms, are becoming increasingly common. For these reasons, neurosyphilis is a diagnostic challenge. Among the diagnostic tests for neurosyphilis, the Venereal Disease Research Laboratory (VDRL) test and the Rapid Plasma Reagin (RPR) test in the cerebrospinal fluid (CSF) are considered the most specific. However, their sensitivity is low [13]. The assessment of the synthesis of TP-specific antibodies in CSF by the Treponema-specific antibody index (TP-AI) is a sensitive method to prove that the bacteria entered the central nervous system (CNS) and produced a local immune reaction. A positive TP-AI can persist after adequately cured infection [8]. Because of the variety of existing and developing techniques for the diagnosis of neurosyphilis, clinical suspicion continues to play the main role [13].

For the reasons outlined, we performed routine testing of patients with cognitive abnormalities or neuropathy for antibodies against TP. We assessed whether, in conjunction with clinical symptoms compatible with syphilis of the nervous system, any laboratory indicators of active syphilis were present in individual patients. When we detected hints of an active TP infection, the patient was treated by antibiotics.

## Patients and methods

A Treponema pallidum electrochemiluminescence immunoassay (TP-ECLIA, Roche Diagnostics, Grenzach-Wyhlen, Germany, normal values < 1.0 arbitrary units [AU]) was performed in all patients with cognitve decline or neuropathy and no or insufficient previous diagnostic workup, who were treated as in-patients at the Dept. of Geriatrics, Protestant Hospital Göttingen-Weende. Patients with cognitive decline were identified by the initial medical examination. Cognitive function was quantified by the Mini Mental Status Test (MMST: abnormal < 27 of 30 scores). In patients with a very high or low level of education, where MMST is unreliable, in addition other appropriate diagnostic tools were used [e.g., parts of the Consortium to Establish a Registry for Alzheimer’s Disease (CERAD) test battery] [14]. Dementia and mild cognitive impairment were differentiated by the test results and the clinical significance of the impairment for everyday life. The cognitive status of patients admitted with delirium of different causes was re-assessed after resolution of delirium. Verbal consent was obtained from the patient or the closest relative to assess the causes of cognitive decline and/or neuropathy including testing for TP-specific antibodies. In patients who or whose relatives refused these differential diagnostic measures, TP-ECLIA was not performed. Patients with elevated TP antibodies as assessed by ECLIA treated from October 2015 to January 2022 (76 months) were included in this retrospective chart review. In cases of positive TP-ECLIA, further specific laboratory investigations were performed to assess the activity of the infection and to determine, whether antibiotic therapy was indicated. For this purpose, the following laboratory tests were performed: 1. Rapid Plasma Reagin test [RPR, modified Venereal Disease Research Laboratory test (VDRL)] (Biorad, Feldkirchen, Germany, normal titers < 1:2), 2. TP-specific IgM enzyme immunoassay (EIA) (Euroimmun, Lübeck, Germany, normal < 16–22 AU/ml), and 3. TP-specific IgG immunoblot (Mikrogen Diagnostik, Neuried, Germany). Furthermore, in 13 patients with a positive TP-ECLIA, the Treponema pallidum particle agglutination assay (TPPA) (Biorad, Feldkirchen, Germany, normal < 1:80) was performed in parallel with the TP-ECLIA. Patients with a positive TP-ECLIA and a positive RPR or TP IgM EIA were treated with antibiotics.

Patients with a positive TP-ECLIA and a positive TP-specific immunoblot, but a negative RPR and no detectable antibodies in the TP IgM EIA were advised to consent into a lumbar puncture for CSF analysis. CSF analysis included a CSF leukocyte count, CSF lactate and total protein concentration, an assesment of the function of the blood-CSF barrier by the determination of the CSF-to-serum albumin ratio, a quantitation of the intrathecal IgG, IgA and IgM synthesis by Reiber-Felgenhauer nomograms [15], and an assessment of the intrathecal synthesis of TP-specific antibodies. For this purpose, serum and CSF antibodies against TP were determined by Enzygnost Syphilis enzyme immunoassay (EIA) (Siemens Healthineers, Erlangen, Germany), and the TP-specific antibody index (TP-AI) was determined. The TP-AI was calculated according to the following equation [16]:

$$\mathrm{Treponema}-\text{specific antibody index }(\mathrm{TP}-\mathrm{AI}) = \frac{\frac{\mathrm{TP}-\text{EIA IgG or IgM in CSF }(\mathrm{U}/\mathrm{ml}) }{\mathrm{TP}-\text{EIA IgG or IgM in serum }(\mathrm{U}/\mathrm{ml})}}{\frac{\text{Total IgG or IgM in CSF }(\mathrm{mg}/\mathrm{l}) }{\text{Total IgG or IgM in serum }(\mathrm{mg}/\mathrm{l})}}$$

TP-AI was determined separately for IgG and IgM. Patients with a positive TP-ECLIA in serum and an increased CSF leukocyte count (> 4 leukocytes/µl) or a positive TP-AI (either IgG or IgM) and no previous adequate antibiotic therapy were also treated with antibiotics.

Because data often were not normally distributed, they were shown as medians (25th/75th percentile). Groups were compared by Kruskal–Wallis and Chi^2^ test. For the assessment of a possible correlation between TP antibodies in TP-ECLIA and TPHA, the non-parametric Spearman`s rank correlation coefficient (r_S_) was used. *P* ≤ 0.05 was considered statistically significant. Statistical analysis was performed by Graph Pad Prism 5.01 (GraphPad Software, Boston, MA 02110, USA).

The patients` data were pseudonymized and then analyzed in a retrospective manner. The study was approved by the Ethics Committee of the University Medicine Göttingen.

## Results

**Fig. 1:**
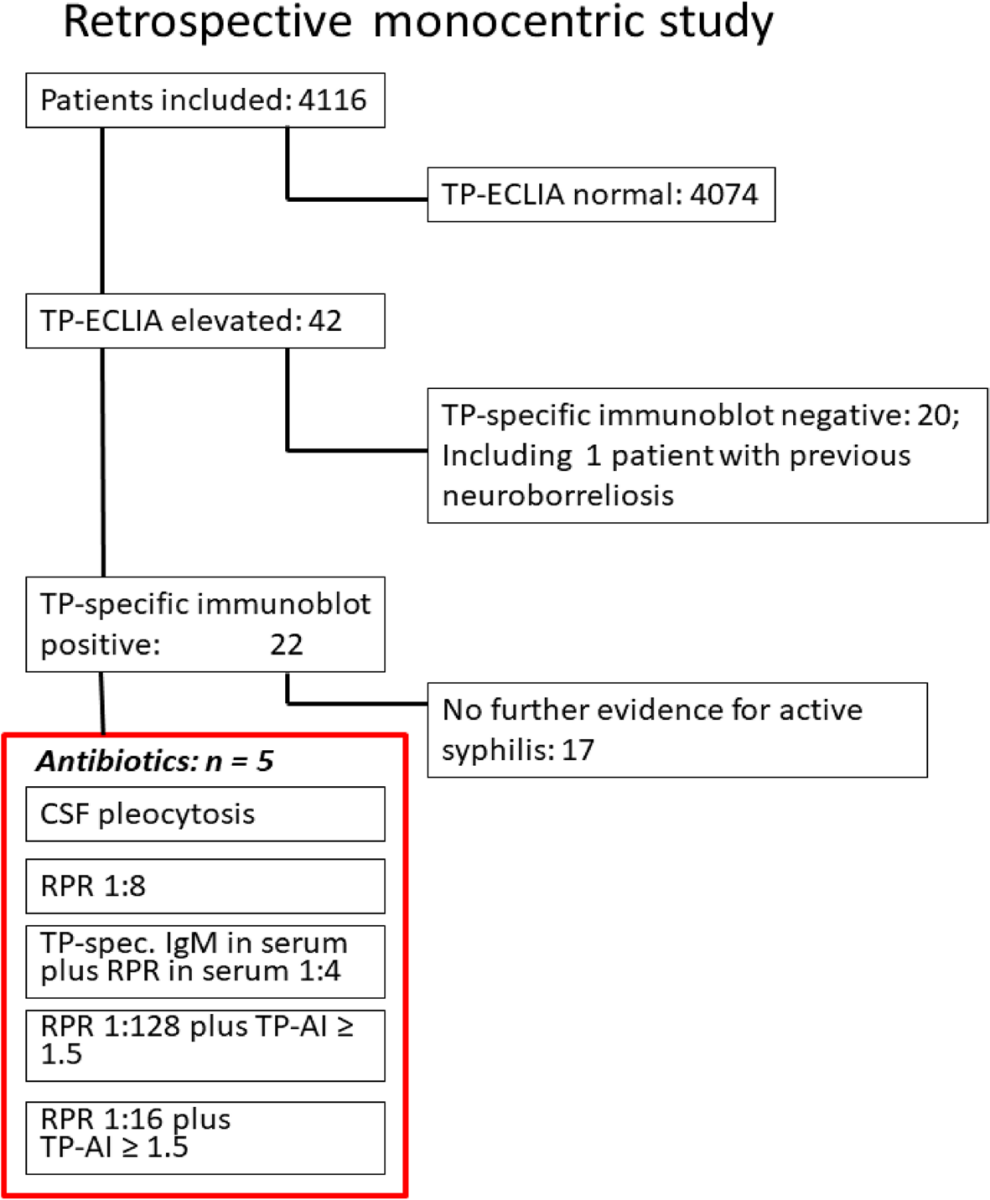
Flow chart. Patients included in the study, patients with elevated Treponema pallidum-specific IgG antibodies determined by electrochemiluminescence immunoassay (TP-ECLIA), and patients with indicators of active infection receiving antibiotics are listed. AI = antibody index; CSF = cerebrospinal fluid; IgM = immunoglobulin M; TP = Treponema pallidum; RPR = Rapid Plasma Reagin test

In 42 of 4116 patients (1.0%, 32 women, 10 men), TP-ECLIA detected antibodies directed against TP in serum (Table 1, Fig. 1). In general, TP-specific antibody concentrations in TP-ECLIA were low (median = 2.75 AU/ml, 25th/75th percentile 1.5/4.88 AU/ml). All patients with detectable TP-specific antibodies in serum had cognitive impairment or distal-symmetric neuropathy. 37 patients with TP-specific antibodies either suffered from mild cognitive impairment or dementia, and 26 patients presented with distal-symmetric neuropathy. 5 patients with a positive TP-ECLIA had only neuropathy. The principal reasons for testing in patients with a positive TP-ECLIA are listed in Table 1. MMST scores were available in 33 patients (median 18, 25th/75th percentile 13.5/24) (normal values ≥ 27).

**Table 1:** Demographic data of the patients studied

Patients	n	Age (years)	Sex (w/m)	Principal reason for TP-ECLIA test		
				Dementia	MCI	Neuropathy
TP-ECLIA negative	4074	85 (80/89)	2526/1548	na	na	na
TP-ECLIA positive, WB negative	20	89 (80/92)	14/6^a^	12	7	1
TP-ECLIA positive, WB positive	22	85 (79/91)	19/3^a^	8	10	4
Antibiotic treatment	5	79 (59/93)	4/1	2	3	0

Data are expressed as medians (25th/75th percentile). The age of the 3 different groups did not differ significantly (*p* = 0.55, Kruskal–Wallis test)

*MCI* Mild cognitive impairment, *TP-ECLIA* Treponema pallidum electrochemiluminescence immunoassay, *WB* Western blot, *na* not analyzed

^a^The frequency of women with a positive TP-ECLIA was higher than the frequency of women among the patients with a negative TP-ECLIA (*p* = 0.04, two-sided Chi^2^ test)

TPPA was applied in addition to TP-ECLIA in 13 patients. In 9 of these patients (69%), TPPA also detected antibodies directed against TP in serum. The titers of TP-specific antibodies detected by TPPA were also low (median 1:80, 25th/75th percentile < 1:80/ 160). There were no patients with a positive TPPA and a negative TP-ECLIA. TP-ECLIA and TPPA titers were not strongly correlated (r_S_ = 0.21, *p* > 0.05). Specifity of the antibodies detected by TP-ECLIA was ensured by immunoblot in 22 patients (11 × positiv, 11 × borderline finding).

RPR test and the measurement of TP IgM EIA were performed in all except one patients with a positive TP-ECLIA (the patient, where RPR test and TP IgM EIA was not performed, had a very low TP-ECLIA value of 1.1). RPR test was positive in 3, and TP-specific IgM was detected by EIA in 1 patient. RPR test and TP IgM EIA were positive in 1 patient, in the other 2 patients RPR test was positive, and TP IgM EIA was negative.

CSF analysis was available in 11 patients (8 CSF samples obtained during the stay at the Dept. of Geriatrics, Protestant Hospital Göttingen-Weende, 3 obtained by other hospital departments). CSF leukocyte counts were elevated (> 4/µl) in 2 patients:

- One man (87 years) with a low TP-ECLIA value of 1.4 AU/ml in serum had a CSF pleocytosis of 190 leukocytes/µl 12 years before his stay in the geriatric department. Then, he was diagnosed with neuroborreliosis (Borrelia-specific AI for IgG 25.5) and treated successfully as documented by a repeat lumbar puncture with a normal CSF leukocyte count 10 months later, i.e., 11 years before the present admission. In this patient, the low TP-ECLIA value (the specifity of the antibodies were not confirmed by Western blot, RPR and TPPA tests were negative, and IgM-specific TP antibodies were not detected) must be considered a cross-reaction in the TP-ECLIA between antibodies directed against Borrelia burgdorferi and TP. Therefore, the patient was not treated with antibiotics.
- In patient #1 (woman, 94 years) with severe dementia, a CSF pleocytosis of 20 leukocytes/µl was found 6.5 years before the present admission, At that time, viral meningitis was assumed, and the patient was treated with aciclovir, but not with an antibacterial drug. Here, TP-specific antibodies were detected in serum: TP-ECLIA (5.7 AU/ml), TPPA (1:160) and TP-IgG Western blot. RPR was negative. Because the patient refused a repeat lumbar puncture and a 14 days stay at our institution, she was treated with doxycyclin 300 mg/d for 15 days.

**Fig. 2:**
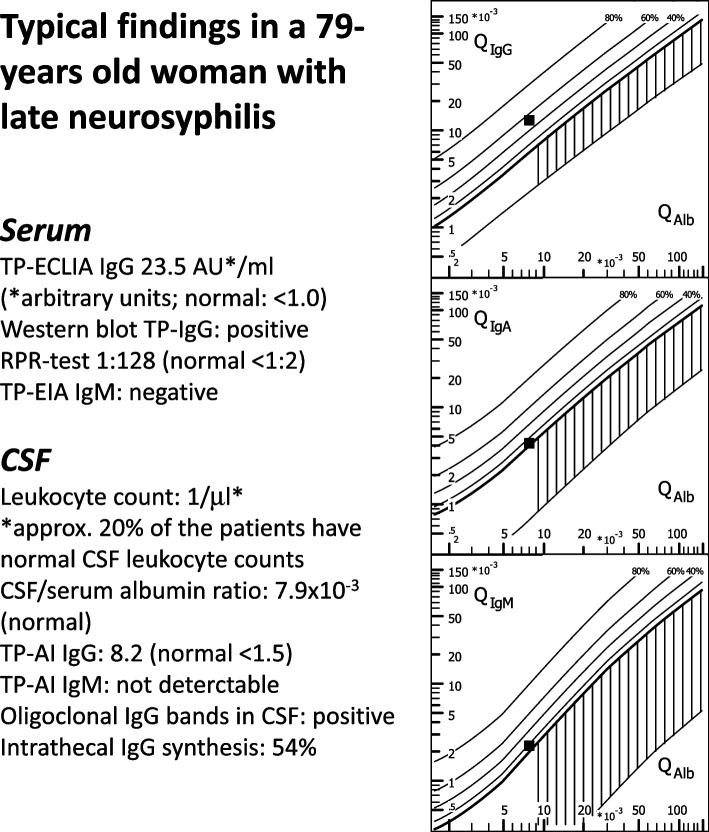
Typical CSF finding in late neurosyphilis (patient #4). Intrathecal synthesis of IgG was demonstrated by 3 methods: Reiber-Felgenhauer nomograms and isoelectric focussing detect overall intrathecal antibody synthesis and are not specific for syphilis, whereas the Treponema pallidum-specific antibody index (TP-AI) of 8.2 proves intrathecal synthesis of TP-specific IgG antibodies. Abbreviations: AI = antibody index; CSF = cerebrospinal fluid; ECLIA = electrochemiluminescence immunoassay; IgG = immunoglobulin G; IgA = immunoglobulin A; IgM = immunoglobulin M; Q_Alb_ = CSF-to-serum albumin quotient; Q_IgG_ = CSF-to-serum IgG quotient; Q_IgA_ = CSF-to-serum IgA quotient; Q_IgM_ = CSF-to-serum IgM quotient; TP = Treponema pallidum; RPR = Rapid Plasma Reagin test. Diagram drawn using the program „Protein Statistics in CSF analysis with Reibergrams” (Albaum IT-Solutions, Göttingen, Germany, Version 4.17, 2012–2013)

The other patients had normal CSF leukocyte counts:

- Patient #2 (woman, 75 years) with a mild cognitive deficit had an elevated TP-ECLIA of 2.2 AU/ml in serum, and RPR was elevated (1:8). CSF leukocytes were normal, and TP-AI was not elevated (IgG 0.72, IgM not detectable). Because of the elevated RPR she received ceftriaxone for 14 days.
- Patient #3 (woman, 92 years) with moderate dementia in the hospital suffered a right-hemispheric cerebral infarction. TP-ECLIA and RPR in serum were elevated (2.1 IU/ml and 1:4), and TP-specific IgM antibodies were strongly elevated (124 AU/ml, normal < 22 AU/ml). CSF analysis revealed a moderate blood-CSF barrier dysfunction (CSF-to-serum albumin ratio 11.6), but no CSF pleocytosis. Because of the elevated TP-specific IgM and the mild RPR elevation she received ceftriaxone for 14 days.
- Patient #4 (woman, 79 years) with a mild cognitive deficit in serum had a TP-ECLIA of 23.5 AU/ml and a RPR of 1:128. Although CSF leukocyte count and CSF-to-serum albumin ratio were not elevated, Reiber-Felgenhauer diagrams and isoelectric focussing revealed an intrathecal synthesis of IgG. IgG TP-AI was elevated (8.2) proving an intrathecal synthesis of TP-specific antibodies, whereas IgM TP-AI was not detectable (Fig. 2). The patient was treated with ceftriaxone for 14 days because of the elevated RPR and IgG TP-AI.

- Patient #5 (man, 43 years, HIV-positive) with a mild cognitive deficit, headache and a syphilis 4 years before admission treated with 3 intramuscular injections of benzathin-penicillin had an elevated TP-ECLIA (23.6 AU/ml) and RPR (1:16) in serum. CSF leukocytes and CSF-to-serum albumin ratio were normal. Isoelectric focussing revealed intrathecal IgG synthesis, whereas Reiber-Felgenhauer nomograms showed no intrathecal immunoglobulin synthesis. IgG TP-AI was elevated (2.9), IgM TP-IA was not detectable. Antibiotic treatment with ceftriaxone for 14 days was administered because of the elevated RPR and IgG TP-AI.

To sum up, as a consequence of positive findings in RPR test, TP IgM EIA or CSF findings (CSF pleocytosis or TP-AI ≥ 1.5), 5 patients received antibiotic therapy. The specific reasons for the initiation of antibiotic therapy were CSF pleocytosis (1 patient), intrathecal TP-specific antibody production as assessed by an elevated TP-specific AI (2), elevated RPR (4) and elevated TP-specific IgM antibodies in serum (1). In order not to immobilize geriatric patients for the i.v. infusion of penicillin G 3 × daily, ceftriaxone 2 g once daily i.v. was used as the standard treatment at our institution. Antibiotic treatment was carried out for at least 14 days. 4 patients received ceftriaxone i.v., and one patient who refused to stay 14 days at our institution was treated with doxycycline per mouth 300 mg/d.

The other 17 patients with a positive TP-ECLIA and a positive or borderline immunoblot together with a negative RPR and no detectable TP-specific IgM antibodies in serum with no CSF pleocytosis or absent CSF analysis were considered not to suffer from active syphilis and were therefore not treated by antibiotics.

## Discussion

TPPA, TP-EIA and TP-ECLIA are highly sensitive assays to detect TP-specific antibodies in body fluids [17, 18]. The sensitivity of TP-EIA and TP-ECLIA appeared to be slightly higher than the sensitivity of TPPA (100% and 100% versus 96% in a recent Chinese study) at a comparable diagnostic efficiency [17]. In the present study, TP-ECLIA was more sensitive than TPPA. Therefore, TPPA was abandoned at our institution in favor of the TP-ECLIA as the routine screening procedure for TP infections.

The prevalence of TP-specific antibodies detected by TP-ECLIA at our institution (1%) was higher than the incidence in the German population estimated from data reported to the Robert Koch Institute (6.5 cases per 100000 standardized person-years in 2010–2012). It was also higher than the seroprevalence for TP-specific antibodies in first-time German blood donors (42.5/100000) [19]. The prevalence of TP-specific antibodies in blood donors in Germany was considerably lower than the prevalence in the United States or less developed countries (summarized by [19]). The pevalence of TP-specific antibodies in serum at our institution was approx. 50% of the prevalence found in a large hospital-based Chinese study [20].

Whereas usually the prevalence of TP-specific antibodies in serum is higher in men than in women, in the present study, 33 of 42 seropositive patients (79%) were female. This is higher than the percentage of women of the number of tested patients in the present study (62%, Table 1) (*p* = 0.04, Chi^2^ test) and of the percentage of women of the number of patients treated at our institution (approx. 63%) [21]. The age of the patients in the present study compared well with a previous retrospective analysis of causes of dementia at our institution (82.9 ± 6.4 years) [22]. As in the present study, an unusually high number of syphilis cases was noted among older women in a German health insurance data set [19]. It was suspected that this phenomenon might be a remnant of the surviving World War II generation [19]. Since TP-specific antibodies can persist over the whole life in spite of effective antibiotic treatment [23], a rising seroprevalence of TP-specific antibodies is not uncommon in aging populations [19, 24, 25].

One patient with previous successfully treated neuroborreliosis had low seemingly TP-specific antibodies in TP-ECLIA, but not in TPPA, and Western blot failed to confirm specificity of these antibodies. Although apparently uncommon [26], positive TP antibody detection assays have been reported in Borrelia burgdorferi infections [27]. Consequently, this patient did not receive antibiotic treatment.

Compared to other bacterial infections, where diagnosis is made by culture or PCR, the diagnosis of syphilis relies on the detection of TP-specific antibodies. As a consequence of the low bacterial load, PCR methods to detect TP DNA from blood or urine are not sensitive enough for clinical routine in late forms of syphilis [28, 29]. The criteria used in the present study to identify patients requiring antibiotic therapy are generally accepted: positive TP-ECLIA plus specificity of antibodies confirmed by Western blot and (positive RPR or TP IgM EIA or CSF pleocytosis or TP-AI ≥ 1.5) [30] (Table 2). Among these criteria, CSF leukocytosis is not very sensitive: 19 of 110 HIV-negative patients (17%) with symptomatic neurosyphilis had a CSF leukocyte count ≤ 5/µl, and 35 of these patients (32%) had a normal CSF protein content [31]. In HIV-positive patients with neurosyphilis, CSF pleocytosis and protein content elevation are even rarer [32]. Conversely, TP-AI is not a good measure of the acuity of a TP infection: in acute infections, pathogen-specific AIs often are not elevated. After successful treatment of a CNS infection, the decline of the pathogen-specific antibodies in serum often is quicker than in CSF. Therefore, after successful treatment of a CNS infections, the pathogen-specific AI can increase, whereas both the serum and CSF concentrations of the pathogen-specific antibodies decrease [33]. Since the use of these activity criteria in ambiguous situations recommend antibiotic treatment, we cannot exclude that in this series one or another case without replication-competent TP was treated by antibiotics.

**Table 2:** Indications for antibiotic treatment in the present study (according to [20, 24])

TP-specific IgG serum antibodies detected by ECLIA		**at least one of the following activity criteria**	
		VDRL or RPR positive	
**plus**	plus	TP-specific IgM positive	
TP-specific IgG antibodies confirmed by Western blot (positive or boderline)		CSF pleocytosis (> 4/µl)	
		TP-specific AI for IgG or IgM ≥ 1.5	**and** no previous antibiotic therapy for neurosyphilis

*AI* antibody index, *CSF* cerebrospinal fluid, *ECLIA* electrochemiluminescence immunoassay, *IgG* immunoglobulin G, *IgM* immunoglobulin M, *TP* Treponema pallidum, *RPR* Rapid Plasma Reagin test, *VDRL* Venereal Disease Research Laboratory test

The standard antibiotic treatment of late neurosyphilis is penicillin G at high doses. Because of its short elimination half-life in serum, it must be administered several times daily. Different treatment protocols are available, most commonly 6 million international units (IU) are infused intravenously (i.v.) 4 × daily, or 5 million IU 5 × daily, or 10 million IU 3 × daily [30, 34]. For geriatric patients, protocols requiring intravenous infusions more frequent than once daily are not desirable, because they promote immobilization of the patient. Moreover, in the elderly with impaired renal function, high-dose penicillin G entails the risk of antibiotic-associated encephalopathy characterized by epileptic seizures and psychosis [35]. For these reasons, our standard treatment protocol consists of ceftriaxone 2 g i.v. once daily for 14 days, which is considered equally effective as high-dose penicillin [34]. Doxycycline administered orally at a daily dose of 300 mg is licensed in Germany for the treatment of syphilis. At high daily doses its physicochemical properties ensure sufficient concentrations in the CNS to treat neurosyphilis [36–38]. Case reports suggest that high-dose doxycycline indeed is effective in neurosyphilis [39]. For this reason, we chose doxycycline in one patient who refused i.v. antibiotic therapy.

The strength of our study is the high number of patients included and the consequent use of TP-ECLIA in the differential diagnosis of dementia at our institution. The main weakness of this study is the lack of a follow-up of the patients treated. Therefore, we are unable to provide data on the success of antibiotic treatment. For the following reasons, we did not attempt to establish a follow-up of the patients treated: a) patients suffering from cognitive decline were not able to reliably report their cognitive status, and b) patients and their relatives were very reluctant to talk about the infection because often they were ashamed of the diagnosis.

## Conclusion

Approx. 1% of German geriatric in-patients with cognitive decline, who received a TP-ECLIA as serological screening test to rule out syphilis, had TP-specific antibodies detected by ECLIA in serum. In 0.5%, the specificity of the antibodies detected by ECLIA was confirmed by Western blotting. In 1‰, either the detection of TP-specific IgM im serum, or a positive RPR in serum, or CSF pleocytosis or a TP-specific intrathecal antibody synthesis ≥ 1.5 required antibiotic treatment with ceftriaxone or doxycycline. As a consequence of the low prevalence of syphilis in geriatric German patients with cognitive decline, from a pharmacoeconomic view the routine testing of all patients with cognitive decline for TP antibodies appears questionable. Since dementia syndromes are highly stigmatizing disease entities, all measures for causal treatment should be utilized. Therefore, we suggest to continue routine testing for syphilis in patients with cognitive decline. Further research with long-term follow up after adequate antibiotic treatment should elucidate, whether these patients with presumable very late neurosyphilis really benefit from antibiotic therapy.

## Data Availability

The datasets used and/or analysed during the current study are available from the corresponding author on reasonable request.

## Abbreviations

AI: Antibody index
CSF: Cerebrospinal fluid
ECLIA: Electrochemiluminescence immunoassay
EIA: Enzyme immunoassay
IgG: Immunoglobulin G
IgM: Immunoglobulin M
RPR: Rapid Plasma Reagin test
TP: Treponema pallidum
TP-AI: Treponema-specific antibody index
TPPA: Treponema pallidum particle agglutination assay
VDRL: Venereal Disease Research Laboratory test

## References

[1] Nitrini R (2005). The cure of one of the most frequent types of dementia: a historical parallel. Alzheimer Dis Assoc Disord.

[2] Nitrini R, de Paiva ARB, Takada LT, Brucki SMD (2010). Did you rule out neurosyphilis?. Dement Neuropsychol.

[3] Bremer V, Dudareva-Vizule S, Buder S, An der Heiden M, Jansen K (2017). Sexually transmitted infections in Germany: the current epidemiological situation. Bundesgesundheitsblatt Gesundheitsforschung Gesundheitsschutz.

[4] Arnold SE, Kumar A (1993). Reversible dementias. Med Clin North Am.

[5] Waldemar G, Dubois B, Emre M, Georges J, McKeith IG, Rossor M, Scheltens P, Tariska P, Winblad B, EFNS (2007). Recommendations for the diagnosis and management of Alzheimer's disease and other disorders associated with dementia: EFNS guideline. Eur J Neurol.

[6] Deuschl G, Maier W (2016). S3-Leitlinie Demenzen der Deutschen Gesellschaft für Neurologie, Version 2.

[7] Ghanem KG (2010). Neurosyphilis: a historical perspective and review. CNS Neurosci Ther.

[8] Prange HW, Moskophidis M, Schipper HI, Müller F (1983). Relationship between neurological features and intrathecal synthesis of IgG antibodies to Treponema pallidum in untreated and treated human neurosyphilis. J Neurol.

[9] Friedrich F, Geusau A, Friedrich ME, Vyssoki B, Pfleger T, Aigner M (2012). The chameleon of psychiatry - psychiatric manifestations of neurosyphilis. Psychiatr Prax.

[10] Tiwana H, Ahmed A (2018). Neurosyphilis: mighty imitator forays with benign presentation and unique neuroimaging findings. Sex Health.

[11] Green J, Savage N, Jenkins C, Chima-Okereke C (2019). Lesson of the month 1: Neurosyphilis mimicking viral encephalitis and ischaemic stroke. Clin Med (Lond).

[12] Murtza M, Bangash A, Rehman AU, Pervaiz A, Imran A (2022). A case of neurosyphilis with psychosis and hippocampal atrophy. Gen Psychiatr.

[13] Boog GHP, Lopes JVZ, Mahler JV, Solti M, Kawahara LT, Teng AK, Munhoz JVT, Levin AS (2021). Diagnostic tools for neurosyphilis: a systematic review. BMC Infect Dis.

[14] Fillenbaum GG, van Belle G, Morris JC, Mohs RC, Mirra SS, Davis PC, Tariot PN, Silverman JM, Clark CM, Welsh-Bohmer KA, Heyman A (2008). Consortium to Establish a Registry for Alzheimer's Disease (CERAD): the first twenty years. Alzheimers Dement.

[15] Reiber H, Felgenhauer K (1987). Protein transfer at the blood cerebrospinal fluid barrier and the quantitation of the humoral immune response within the central nervous system. Clin Chim Acta.

[16] Jacobi C, Lange P, Reiber H (2007). Quantitation of intrathecal antibodies in cerebrospinal fluid of subacute sclerosing panencephalitis, herpes simplex encephalitis and multiple sclerosis: discrimination between microorganism-driven and polyspecific immune response. J Neuroimmunol.

[17] Liu C, Ou Q, Chen H, Chen J, Lin S, Jiang L, Yang B (2014). The diagnostic value and performance evaluation of five serological tests for the detection of Treponema pallidum. J Clin Lab Anal.

[18] Lee JH, Lim CS, Lee MG, Kim HS (2015). Evaluation of a rapid immunochromatographic treponemal antibody test comparing the Treponema pallidum particle agglutination assay. J Clin Lab Anal.

[19] Šmit R, Wojtalewicz N, Vierbaum L, Nourbakhsh F, Schellenberg I, Hunfeld KP, Lohr B (2022). Epidemiology, management, quality of testing and cost of syphilis in Germany: a retrospective model analysis. Front Public Health.

[20] Xu K, Chi S, Chen B, Chen L, Zheng D (2016). The distribution of syphilis among inpatients in Wenzhou, China: a hospital based study. Jundishapur J Microbiol.

[21] Dohrendorf CM, Unkel S, Scheithauer S, Kaase M, Meier V, Fenz D, Sasse J, Wappler M, Schweer-Herzig J, Friede T, Reichard U, Eiffert H, Nau R, Seele J (2021). Reduced Clostridioides difficile infections in hospitalised older people through multiple quality improvement strategies. Age Ageing.

[22] Djukic M, Wedekind D, Franz A, Gremke M, Nau R (2015). Frequency of dementia syndromes with a potentially treatable cause in geriatric in-patients: analysis of a 1-year interval. Eur Arch Psychiatry Clin Neurosci.

[23] Gottlieb SL, Pope V, Sternberg MR, McQuillan GM, Beltrami JF, Berman SM (2008). Prevalence of syphilis seroreactivity in the United States: data from the National Health and Nutrition Examination Surveys (NHANES) 2001–2004. Sex Transm Dis.

[24] Jitapunkul S (2000). Syphilitic seroreactivity among the Thai population aged 50 years and above: value of mass screening. Southeast Asian J Trop Med Public Health.

[25] Wu X, Guan Y, Ye J, Fu H, Zhang C, Lan L (2019). Association between syphilis seroprevalence and age among blood donors in Southern China: an observational study from 2014 to 2017. BMJ Open.

[26] Patriquin G, LeBlanc J, Heinstein C, Roberts C, Lindsay R, Hatchette TF (2016). Cross-reactivity between Lyme and syphilis screening assays: Lyme disease does not cause false-positive syphilis screens. Diagn Microbiol Infect Dis.

[27] Enders G, Biber M, Baier R, Hlobil H, Wellensiek HJ (1988). Suspected syphilis during pregnancy due to cross reactions in Borrelia infection. Dtsch Med Wochenschr.

[28] Zhang Y, Dai X, Ren Z, Lin H, Cao W, Ye X (2019). A novel nested real-time polymerase chain reaction for Treponema pallidum DNA in syphilis biospecimens. Sex Transm Dis.

[29] Wang C, Zheng X, Guan Z, Zou D, Gu X, Lu H, Shi M, Zhou P (2022). Quantified detection of Treponema pallidum DNA by PCR assays in urine and plasma of syphilis patients. Microbiol Spectr.

[30] Klein M, Angstwurm K, Esser S, Hahn K, Maschke M, Scheithauer S, Schoefer H, Sturzenegger M, Wildemann B, Weber J (2020). German guidelines on the diagnosis and treatment of neurosyphilis. Neurol Res Pract.

[31] Li W, Jiang M, Xu D, Kou C, Zhang L, Gao J, Qin K, Wu W, Zhang X (2019). Clinical and laboratory characteristics of symptomatic and asymptomatic neurosyphilis in HIV-negative patients: a retrospective study of 264 cases. Biomed Res Int.

[32] Wang Z, Liu L, Shen YZ, Zhang RF, Qi TK, Tang Y, Song W, Chen J, Lu H (2018). The clinical and laboratory features of neurosyphilis in HIV-infected patients: a retrospective study in 92 patients. Medicine (Baltimore).

[33] Ratzka P, Schlachetzki JC, Bähr M, Nau R (2006). Varicella zoster virus cerebellitis in a 66-year-old patient without herpes zoster. Lancet.

[34] Workowski KA, Bolan GA, Centers for Disease Control and Prevention (2015). Sexually transmitted diseases treatment guidelines, 2015. MMWR Recomm Rep.

[35] Bhattacharyya S, Darby RR, Raibagkar P (2016). Antibiotic-associated encephalopathy. Neurology.

[36] Yim CW, Flynn NM, Fitzgerald FT (1985). Penetration of oral doxycycline into the cerebrospinal fluid of patients with latent or neurosyphilis. Antimicrob Agents Chemother.

[37] Karlsson M, Hammers S, Nilsson-Ehle I, Malmborg AS, Wretlind B (1996). Concentrations of doxycycline and penicillin G in sera and cerebrospinal fluid of patients treated for neuroborreliosis. Antimicrob Agents Chemother.

[38] Nau R, Sörgel F, Eiffert H (2010). Penetration of drugs through the blood-cerebrospinal fluid/blood-brain barrier for treatment of central nervous system infections. Clin Microbiol Rev.

[39] Kang-Birken SL, Castel U, Prichard JG (2010). Oral doxycycline for treatment of neurosyphilis in two patients infected with human immunodeficiency virus. Pharmacotherapy.

